# Influence of Structure Development on Performance of Copper Composites Processed via Intensive Plastic Deformation

**DOI:** 10.3390/ma16134780

**Published:** 2023-07-02

**Authors:** Radim Kocich, Petr Opěla, Martin Marek

**Affiliations:** 1 Faculty of Materials Science and Technology, VŠB–Technical University of Ostrava, 17. Listopadu 2172/15, 70800 Ostrava-Poruba, Czech Republic; 2 Department of Technical Studies, College of Polytechnics Jihlava, Tolsteho 16, 58601 Jihlava, Czech Republic

**Keywords:** copper, oxide dispersion, composite, rotary swaging, microstructure

## Abstract

Designing a composite, possibly strengthened by a dispersion of (fine) oxides, is a favorable way to improve the mechanical characteristics of Cu while maintaining its advantageous electric conductivity. The aim of this study was to perform mechanical alloying of a Cu powder with a powder of Al_2_O_3_ oxide, seal the powder mixture into evacuated Cu tubular containers, i.e., cans, and apply gradual direct consolidation via rotary swaging at elevated temperatures, as well as at room temperature (final passes) to find the most convenient way to produce the designed Al_2_O_3_ particle-strengthened Cu composite. The composites swaged with the total swaging degree of 1.83 to consolidated rods with a diameter of 10 mm were subjected to measurements of electroconductivity, investigations of mechanical behavior via compression testing, and detailed microstructure observations. The results revealed that the applied swaging degree was sufficient to fully consolidate the canned powders, even at moderate and ambient temperatures. In other words, the final structures, featuring ultra-fine grains, did not exhibit voids or remnants of unconsolidated powder particles. The swaged composites featured favorable plasticity regardless of the selected processing route. The flow stress curves exhibited the establishment of steady states with increasing strain, regardless of the applied strain rate. The electroconductivity of the composite swaged at elevated temperatures, featuring homogeneous distribution of strengthening oxide particles and the average grain size of 1.8 µm^2^, reaching 80% IACS (International Annealed Copper Standard).

## 1. Introduction

The majority of commercially produced metallic materials are fabricated by casting, although techniques such as powder metallurgy (PM) [1,2,3], sintering (of various types) [4,5,6], mechanical alloying [7,8,9], and additive manufacturing [10,11,12] are also favored. Fabricating a bulk material from initial powders is advantageous as it supports the achievement of fine grain size and thus provides the materials with improved properties [13,14]. An advantage is also that powders of various materials featuring different properties can be mixed together in a single production step [15,16]. Among the typical disadvantages is, however, the presence of pores within most final cold-sintered components. Residual porosity (negatively) affects the tensile and fracture properties and ductility, imparts brittleness, and influences crack propagation within the produced components [17,18,19,20]. Sintering at elevated temperatures can be implemented to eliminate/reduce the residual porosity [21,22]. Nevertheless, sintering at high temperatures tends to increase the grain size. For this reason, direct consolidation via methods of plastic deformation is favored to achieve a smaller grain size and enhanced properties [23,24]. Furthermore, powder-processing techniques can be combined with plastic deformation. For example, Huang et al. [25] studied the effects of plastic deformation on the performance of a FeNi high entropy alloy fabricated from original powders and reported the final grain size of ~6 µm.

Both conventional (e.g., forging, rolling, extrusion, etc. [26,27,28,29,30]), as well as unconventional (e.g., severe plastic deformation (SPD) methods [31,32,33,34,35,36,37,38] or rotary swaging [39,40,41,42]) plastic deformation techniques can be used to enhance the properties and modify the shape/geometry of powder-based components. Rotary swaging is an industrially applicable method favored for providing the processed materials with both grain size refinement and improvement of properties [43]. During swaging, the imposed small shear strain increments gradually refine the grain size via repeating the chain of grain fragmentation-substructure formation-grain nucleation. Given by its advantageous stress state and incremental character, swaging can be used to process challenging materials (e.g., composites of various types [44,45,46,47,48], materials with low plasticity, challenging and hardenable alloys [49,50,51,52,53,54], materials strengthened with oxide dispersions (ODS) [55,56,57], etc.). Swaging can also be used to process powder-based pre-sintered or additively manufactured workpieces to reduce the residual porosity, improve density, and enhance properties [11]. It is also advantageous for direct consolidation of powder-based materials, including composites, or powder-based materials with dispersions of (oxide) particles [58].

Pure Cu exhibits excellent electroconductivity, but (very) low mechanical properties [59]. Additions of alloying elements usually result in the formation of strengthening precipitates, but deteriorations in electroconductivity. For example, combining Cu with Al typically results in strengthening via formation of the GP (Guinier Preston) zones [60,61]. Adding small amounts of elements known for their strengthening ability can be favored, too; adding Ti imparts the formation of the α-Cu_4_Ti phase [62]. Similarly, additions of Be or Hf provide strengthening via the formation of the γ-CuBe phase [63], or Cu_51_Hf_14_ and Cu_5_Hf precipitates [64]. Another solution is to directly add particles, such as carbon nanotubes [65] or SiC (nano)particles [66], into the matrix to fabricate composites with enhanced strength.

Oxygen has a specific action on Cu-based materials. When dissolved within the structure, it tends to deteriorate the electroconductivity [59]. On the other hand, controlled additions of oxygen in the form of fine precipitates, i.e., strengthening oxide particles, can lead to improvements in the mechanical properties. For example, the presence of Cu_2_O precipitates typically deteriorates plasticity and durability of Cu-based alloys and can result in cracking when in contact with water (hydrogen) [67]. Nevertheless, if optimized, the presence of Cu_2_O precipitates can enhance certain parameters. For example, Jeyaprakash et al. [68] found that the presence of Cu_2_O nanoprecipitates within an additively manufactured Cu-Cr-Zr alloy significantly contributed to grain size refinement, hardness increase, and enhancement of machining behavior. Some researchers studied the effects of additions of Al_2_O_3_ particles. Rajkovic et al. [69] applied hot compression at 800 °C with the time dwell of several hours to compact mixtures of Cu + Al_2_O_3_ powders; although the mechanical properties were quite favorable, the measured electroconductivity was low (~50% IACS). Moustafa et al. [66] used cold consolidation followed by sintering at 900 °C to prepare Cu + Al_2_O_3_ composites, but they primarily focused on studying the mechanical properties and did not perform a correlation with the electric ones. Feng et al. [70] fabricated a Cu + Al_2_O_3_ + SiC whiskers powder-based composite by cold isostatic pressing and sintering at 900 °C; although the achieved electroconductivity was 82% IACS, there was a strong anisotropy related to the orientations of the SiC whiskers within the Cu matrix.

Oxide-dispersion-strengthened (ODS) Cu materials are worth studying, especially in regards to the correlation of mechanical and electric properties. The available studies mostly involve time- and cost-consuming multi-step procedures featuring high temperatures and long time dwells. The performed background research gave rise to the following hypothesis: direct consolidation of powder-based Cu ODS composites via intensive plastic deformation at moderate/low temperatures can provide materials featuring very fine (possibly ultra-fine) grains with (homogeneous) dispersions of strengthening oxide particles, and thus favorable mechanical properties. The aim of the herein presented study was thus to characterize the effects of direct consolidation performed via rotary swaging at various temperatures on structure, electric properties, and mechanical behaviors of a Cu-based ODS composite. The composite was prepared by mechanical alloying of Cu and Al_2_O_3_ powders.

## 2. Materials and Methods

### 2.1. Experimental Material

**Figure 1:**
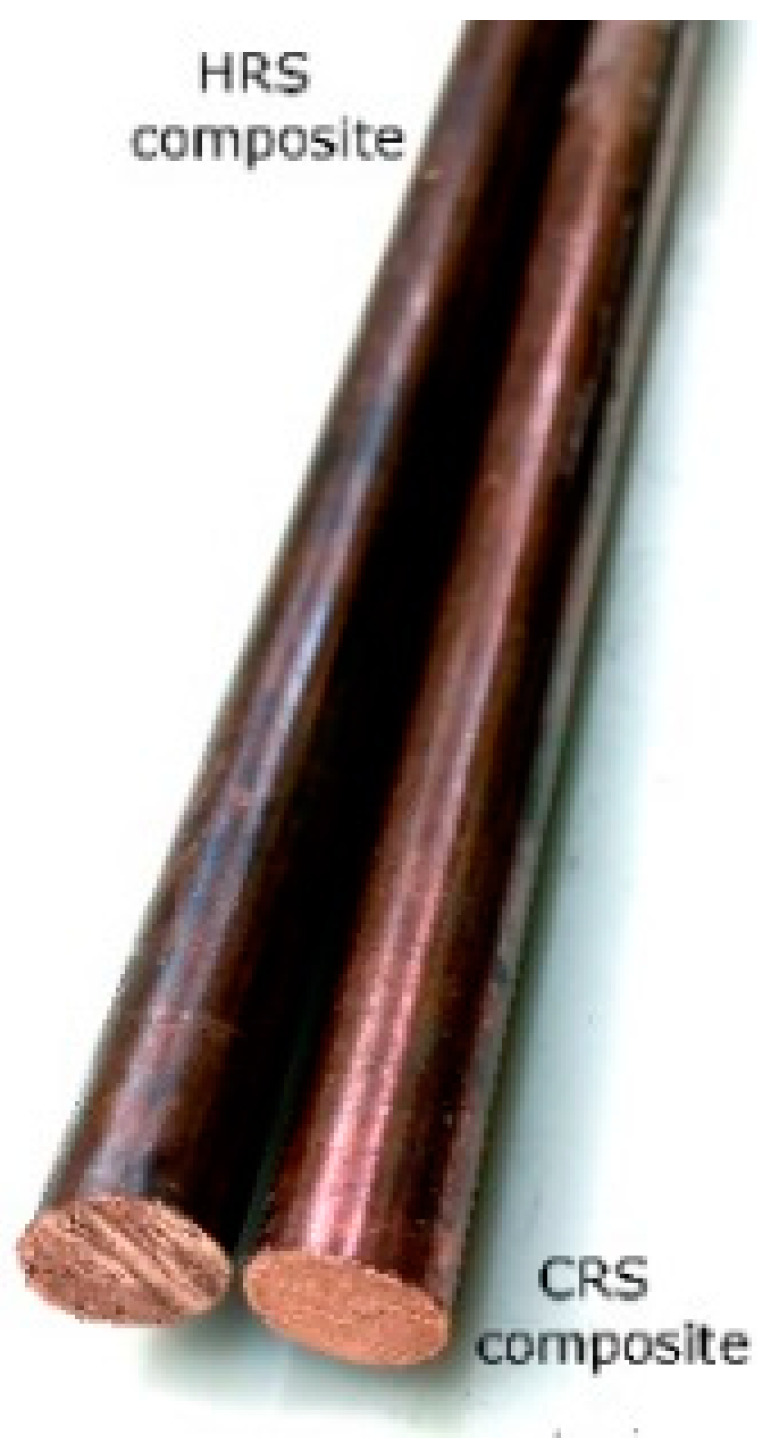
Bars of swaged Cu + Al_2_O_3_ composite.

At the beginning of the experimental works, powders of Al_2_O_3_ (5 wt. %) and Cu (bal.) were subjected to mechanical alloying (MA) for two hours. The size distributions of the original powder particles were the following: 1–10 µm for the Al_2_O_3_ oxide particles and 10–40 µm for the Cu powder particles. The MA time was selected with the intention of making the entire processing procedure as effective as possible. Rajkovic et al. [69] documented that the first five hours of milling have the most substantial effect on fragmentation of the powders and their homogenization. Subsequently, the powder mixture was filled into cans made from Cu tubes with the initial diameter of 25 mm and vacuum sealed. The cans prepared for the deformation processing were pre-heated in a furnace to the initial processing temperature of 400 °C and directly consolidated via gradual rotary swaging. The swaging was performed in six consequent passes from the initial diameter of 25 mm to the final diameter of 10 mm. During the swaging to a diameter of 15 mm, the cans were kept in the furnace heated to the temperature of 400 °C between the individual swaging passes. The final two swaging passes, i.e., from the diameter of 15 mm to the final diameter of 10 mm, were performed differently. For the composite further denoted as *HRS* (i.e., hot rotary swaged), the two final passes were performed at the elevated temperature of 600 °C. On the other hand, the composite further denoted as *CRS*, (i.e., cold rotary swaged), was further swaged at room temperature. Both the swaging procedures were successful, see Figure 1 for the swaged composite bars.

The total swaging degree, i.e., the total imposed strain, calculated using Equation (1) (e.g., [71,72]):

(1) $$\phi =\ln\left(\frac{S_{0}}{S_{n}}\right)$$

where *S*_0_ and *S_n_* are cross-sectional areas of the swaged composite at the input and output of the dies, respectively, was 1.83.

### 2.2. Structure Analyses

Samples cut cross-sectionally from the consolidated bars were subjected to scanning electron microscopy (SEM) analyses of chemical composition (the Energy Dispersive Spectroscopy, i.e., EDX method) and microstructure (the Electron Backscatter Diffraction, i.e., EBSD method). A sample from the composite swaged at the deformation temperature of 400 °C to the diameter of 15 mm and was briefly examined to assess the effects of the imposed strain and processing temperature on the degree of powder consolidation with a greater depth. The structure analyses were carried out using SEM (Tescan Fera3 device, Tescan Orsay Holding a.s., Brno, Czech Republic). The samples for the analyses were prepared by manual grinding using SiC papers with the coarseness set up to 2000, followed by manual polishing using an alcohol-based diamond solution with the particle coarseness of 3 µm and 1 µm. Finally, the samples were polished electrolytically in a solution of ethanol and hydrochloric acid for a time of 40 s (all agents by Struers GmbH, Roztoky u Prahy, Czech Republic). To acquire detailed structure scans for the subsequent evaluations, the used scan step was 0.05 µm. The limiting values considered for the structure evaluations were 15° for full grain, i.e., high angle grain boundary (HAGB), and 5° for a subgrain, i.e., low angle grain boundary (LAGB). The analyses of ideal grains’ orientations, i.e., texture, were performed with a maximum deviation from the ideal orientation of 15°.

### 2.3. Deformation Behavior

The deformation behavior of the presented Cu-based composite can be expected to be unique, as it was fabricated from initial powders by a direct consolidation via intensive plastic deformation. Moreover, it is a composite consisting of Cu and Al_2_O_3_ particles. For this reason, we performed thorough investigations to characterize the behavior of the directly consolidated composite presented.

The deformation behavior of the studied composites processed at both the hot and cold conditions (i.e., *HRS* and *CRS* samples, respectively), was examined by means of a series of uniaxial compression tests—see the characterization of the testing conditions and studied states in Table 1. Note that the study was supplemented with the examinations of conventional electroconductive commercially pure (CP), which was swaged in an identical manner as the *CRS* composite sample, i.e., from the original diameter of 25 mm to the final diameter of 10 mm under warm (400 °C) + cold (~20 °C) conditions. The compression tests were carried out using a Gleeble 3800 thermo-mechanical simulator equipped with a Hydrawedge II mobile conversion testing unit (both by Gleeble, Poestenkill, UK). The cylindrical samples for the compression tests had the diameter of 10 mm and length of 15 mm. During the testing of each sample, the sample-anvils interfaces were separated by tantalum foils in combination with a nickel-based grease to reduce the anvil wear and suppress the friction forces. The testing procedure for both the CP Cu and Cu + Al_2_O_3_ composite was applied at room temperature and various strain rates (Table 1), which resulted in nine flow curves embodying the flow stress evolutions of the examined materials. Each curve was assembled on the basis of the acquired force *F* (N) (gained by a load cell) and absolute deformation Δ*l* (mm) (anvil displacement measured by a linear variable differential transformer transducer). The calculation of the true strain *ε* (-) and true flow stress *σ* (MPa) values was then given as follows (see Equations (2) and (3), respectively):

(2) $$\varepsilon =\ln\left(\frac{l_{0}-\Delta l}{l_{0}}\right)$$

(3) $$\sigma =\frac{F}{\frac{\frac{\pi \cdot d_{0}^{2}}{4}\cdot l_{0}}{l_{0}-\Delta l}}$$

where *d*_0_ (mm) and *l*_0_ (mm) correspond to the initial sample diameter and initial sample length, respectively. Note that the maximal true strain value (Table 1) led to the final sample length of 5.5 mm, which corresponds to the total engineering strain of 63%.

### 2.4. Evaluation of Electroconductivity

The electroconductivity of the Cu + Al_2_O_3_ consolidated composites during the direct current (DC) transfer was measured using the probe of a SIGMATEST 2.070 measuring tool (FOERSTER TECOM s.r.o, Prague, Czech Republic), which is a high-tech eddy current portable equipment enabling direct measurement of the electric resistivity and further assessment of the electroconductivity. The SIGMATEST 2.070 equipment is advantageous for the determination of the electric characteristics of samples acquired from challenging materials, such as the herein presented directly consolidated composite, and can also be used to measure samples with small dimensions. Firstly, calibration of the equipment (i.e., the probe) using two default specimens with default electroconductivities was performed. Then, the probe was used to measure the electroconductivities of the consolidated composites. The data were acquired from the directly consolidated composite rods with the length of 500 mm.

## 3. Results and Discussion

### 3.1. Structure Evaluation

The microstructure characterization focused on evaluating the quality of consolidation of the Cu + Al_2_O_3_ powders, on the grain size and their orientations, and possible differences imparted by the selected processing routes. As the rotary swaging process is based on imposing shear strain into the processed material from its periphery towards its axis, processed workpieces can possibly feature an inhomogeneity of strain distribution across the cross-section, especially for lower applied swaging degrees. For this reason, the microstructures were scanned in the mid-radius distance between the periphery and axis of a particular sample.

The acquired scans were not cleaned before the evaluations to prevent unintentional distortion of the data.

**Figure 2:**
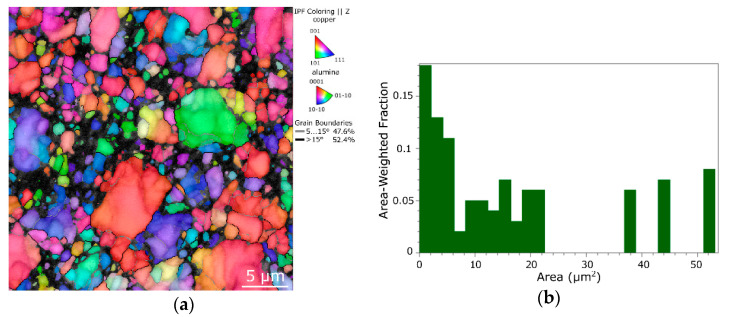
OIM image for structure of 15 mm directly consolidated composite (**a**), corresponding grain size distribution chart (**b**).

To document the structure changes imparted by the differentiated swaging routes with a higher clarity, as well as to further enable deeper discussion and characterization of the consolidated 10 mm Cu + Al_2_O_3_ composites, the structure of the composite swaged to a diameter of 15 mm at a temperature of 400 °C was also examined. The corresponding orientation image map (OIM) is depicted in Figure 2a. As can be seen, the structure was quite heterogeneous and consisted of small, fragmented particles, as well as larger consolidated grains. The grains exhibited a tendency to form the (111) || swaging direction (SD) preferential texture fiber. Given by a relatively high amount of the imposed shear strain, residual porosity, considerable portion of difficult-to-be-detected fine fragments of particles, and presence of oxide particles, the hit rate for this sample, i.e., percentage of analyzed points that were indexed by the microscope, was rather low (72.3%). The grain size distribution chart for the structure is depicted in Figure 2b; evidently, the largest grains reached over 50 µm^2^. The average grain size was 2.5 µm^2^.

**Figure 3:**
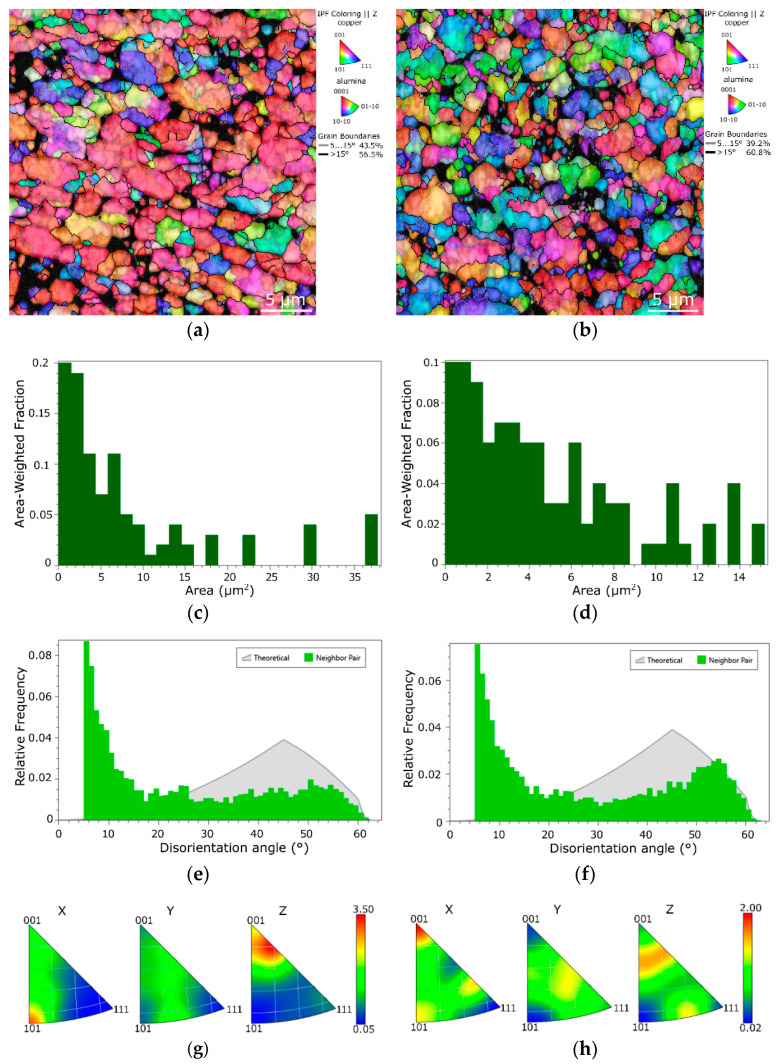
OIM images of the structures of *HRS* (**a**) and *CRS* (**b**) samples. Grain size distribution charts for *HRS* (**c**) and *CRS* (**d**) samples. Grain boundary disorientation angle distribution for *HRS* (**e**) and *CRS* (**f**) samples. IPF maps depicting textures within *HRS* (**e**) and *CRS* (**f**) samples.

Figure 3a,b show the OIM images acquired from the structures of the *HRS* and *CRS* samples of composites swaged to the final diameter of 10 mm. The hit rate for the *HRS* sample was 88.2%, and for the *CRS* it was 85.5%. The non-indexed locations predominantly corresponded to the locations, the oxide particles in which were found (as further documented in Section 3.2).

As can be seen, both the *HRS* and *CRS* structures consisted of fully consolidated Cu grains. The average grain area for the *HRS* sample was 1.8 µm^2^, while for *CRS* it was as low as 1.2 µm^2^ (see Figure 3c,d depicting the area-weighted grain size distribution charts). In other words, the composite of the last two swaging passes for which were performed at room temperature (*CRS*) featured a smaller final grain size. Note also that the maximum grain size within the *HRS* sample reached 35 µm^2^, while for the *CRS* sample, the grain size was more uniform and the largest grains reached only 16 µm^2^. On the other hand, the *HRS* sample exhibited more developed substructure, the notion of which is evident from the shadings of the individual grains in the OIM image in Figure 3a, as well as from the slightly higher LAGB fraction within the structure (the disorientation angle distributions for the *HRS* and *CRS* samples, respectively, are depicted in Figure 3e,f). The final passes performed at room temperature thus evidently caused a higher extent of fragmentation of the consolidated grains. It can be supposed that the effect of the processing temperature in the final two swaging passes was crucial for the (sub)structure development. During the final swaging, the structure of the *HRS* sample processed under the elevated temperature exhibited substructure development, whereas the structure of the *CRS* sample processed at room temperature exhibited grains fragmentation as the plastic flow was aggravated by the decreased processing temperature in the last two swaging passes [73].

The grains’ orientations were also more randomized within the structure of the *CRS* composite than within the structure of the *HRS*, the entire swaging procedure for which was performed under warm + hot conditions. This is obvious not only from the OIM images (Figure 3a,b), but also from the inverse pole figures (IPFs) depicted in Figure 3g,h showing preferential texture fibers within the individual composites. The textures of both the final swaged composites exhibited the tendency to form the (111) || SD preferential fiber, however, without any strikingly high intensity (primarily because the composites were consolidated from original powders and thus the initial state featured no significant preferential texture, see also Figure 2a). Nevertheless, the maximum texture intensity of the *HRS* composite was almost twice as high as that of the *CRS* one. This finding can primarily be attributed to the occurring grain fragmentation and related randomization of the orientations of the grains during the final swaging passes performed at room temperature.

**Figure 4:**
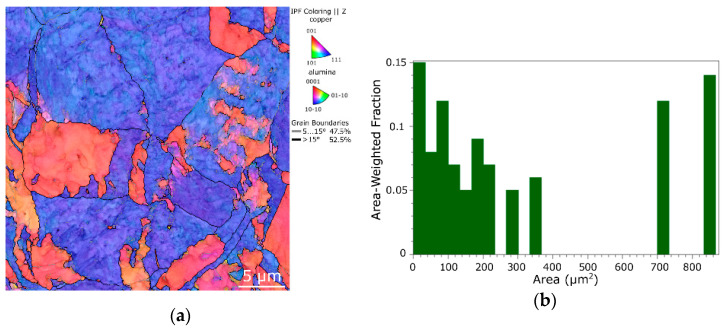
OIM image for structure of 10 mm swaged CP Cu (**a**), corresponding grain size distribution chart (**b**).

As the deformation behaviors of both the 10 mm directly consolidated composites were further compared to that of swaged electroconductive commercially pure (CP) Cu, the structure state of the CP Cu swaged with a procedure identical to that applied for the *CRS* composite was also examined. The OIM image of the swaged CP Cu structure and corresponding grain size distribution chart are depicted in Figure 4a,b. The structure of the swaged CP Cu evidently exhibited a preferential (111) || SD texture fiber, which was in contrast to the composites directly consolidated from the initial powders, which evidently exhibited more randomized grain orientations (compare Figure 4a to Figure 3a,b). Also, the grain size within the swaged CP Cu was incomparably larger; the average grain area was 30.5 µm^2^ and the greatest grains reached up to more than 800 µm^2^.

### 3.2. Oxide Distribution

**Figure 5:**
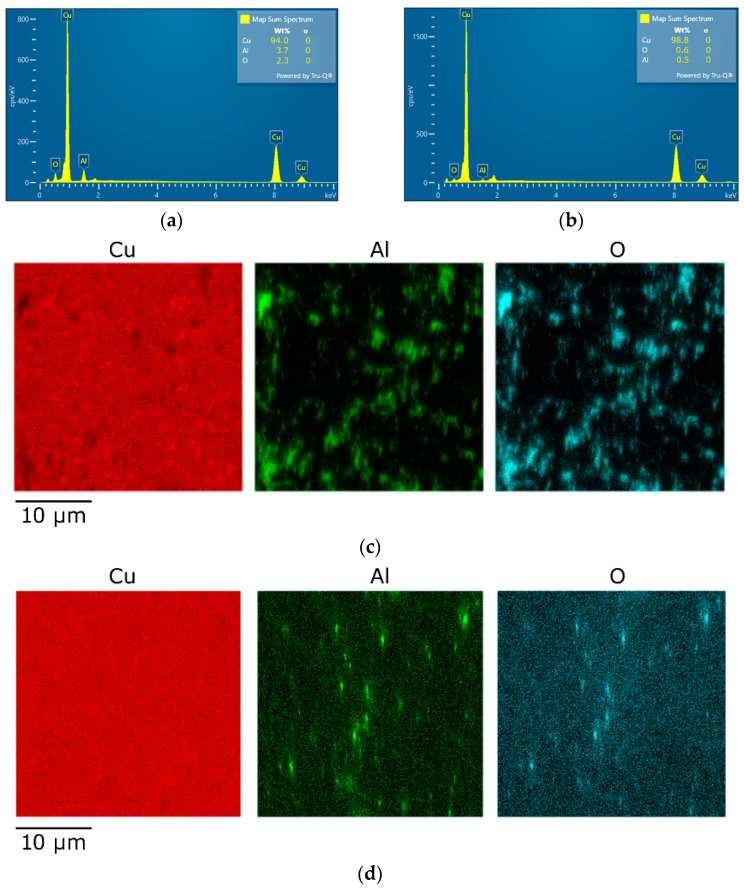
Results of EDX analyses: summary of chemical composition for *HRS* sample (**a**), *CRS* sample (**b**), maps of elements across scanned area for *HRS* sample (**c**), *CRS* sample (**d**).

The performed microscopy analyses also involved the characterization of the overall chemical composition of the 10 mm consolidated composites by the EDX method, as well as evaluation of the dispersion of oxide particles within their structures. Figure 5a shows a graphical depiction summarizing the chemical composition of the *HRS* composite, while Figure 5b shows a graphical depiction summarizing the chemical composition of the *CRS* one. Detailed maps depicting the distributions of the characteristic elements, i.e., Cu, Al, and O, throughout the scanned areas of 30 × 30 µm^2^, from which the above characterized structure analyses were evaluated (Section 3.1), are shown in Figure 5c (*HRS* sample) and 5d (*CRS* sample). In other words, both the EBSD and EDX data sets were collected simultaneously during a single measurement. Therefore, the measured locations within the samples match exactly (the structure in Figure 3a corresponds to Figure 5c, and the structure in Figure 3b corresponds to Figure 5d).

As can be seen in Figure 5a, the *CRS* composite structure contained an overall 94 wt. % of Cu and about 6 wt. % of the Al_2_O_3_ oxide, which corresponds to the desired chemical composition. On the other hand, the measured chemical composition of the *CRS* sample showed more than 98 wt. % content of Cu (Figure 5b). This fact could hypothetically be attributed to the two following factors: (i) the examined *CRS* composite structure contained a lower fraction of the oxide particles, i.e., the structure was less homogeneous than that of the *HRS* composite—this supposition is highly improbable as the cans were both filled from a single mechanically alloyed batch of powder; (ii) by the effect of the final cold processing, the oxide particles fragmented, as also did (to a greater extent, see Figure 3d) the Cu particles; therefore, the size of the majority of the fragments of the oxide particles within the *CRS* composite was below the available resolution of the EBSD method—this supposition is more probable, and is also supported by the EDX mapping, the results of which are shown in Figure 5c,d. Evidently, the oxide particles within the scanned area were finer and more fragmented within the *CRS* sample (Figure 5d) than within the *HRS* one (Figure 5c). The comparison of Figure 5c,d with Figure 3a,b also confirms that the majority of unindexed regions on the EBSD maps corresponded to the locations where the oxide particles were present.

### 3.3. Deformation Behavior

**Figure 6:**
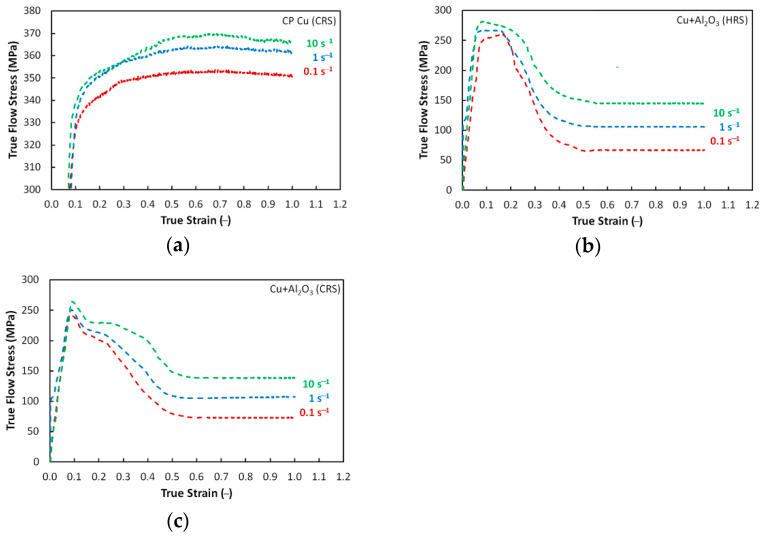
Deformation behavior of rotary swaged CP Cu (**a**), and directly consolidated composites, *HRS* (**b**) and *CRS* (**c**) samples.

The experimentally acquired curves of the evolutions of flow stress in relation with true strain and strain rate for the swaged CP Cu and *HRS* and *CRS* samples of the directly consolidated Cu + Al_2_O_3_ composites are graphically depicted in Figure 6a–c. As can be seen from the Figures, the dependencies were characterized with a typical initial flow stress increase with increasing strain rate, regardless of the initial chemical composition and structure state. However, this was the only observed similarity between the swaged CP Cu and consolidated composites. Otherwise, their deformation behaviors were different.

The addition of the Al_2_O_3_ powder to the Cu powder evidently resulted in a substantial overall flow stress decrease; significant changes were also observed in regards to the trends of the curves (compare Figure 6a to Figure 6b,c). The CP Cu exhibited a simple power-law flow stress increase [74] (Figure 6a), while the flow stress trends of both the *HRS* and *CRS* composites were more complicated; see that the sharp flow stress increase was directly followed by a substantial decrease and subsequent transition to a steady state for both the composite samples (Figure 6b,c). The observed flow stress behaviors of the *HRS* and *CRS* composites gives rise to the hypothesis that regardless of the deformation temperature, the consolidated composite featured enhanced plasticity and thus improved formability (based on the flow stress decrease and establishment of a steady state) when compared to the conventional CP Cu. This finding can primarily be attributed to the differences in the structure states of the examined swaged CP Cu, and directly consolidated composites. Firstly, the examined CP Cu featured a different grain size (see Figure 4a,b), i.e., the grains within the swaged Cu were refined, but still far greater than the grains within the swaged composites, which featured UFG (ultra-fine grained) structures with the majority of sub-micron sized grains (especially the *CRS* one, see Figure 3a–d). When loaded with a critical stress level, the UF grains most probably exhibited grain boundary sliding, which contributed to a decrease in the flow stress and establishment of a steady state [75]. The presence of the oxide particles probably contributed to this phenomenon, as they act as barriers for the movement of dislocations and grain boundaries, which supported grain fragmentation [59]. By this effect, the grains were further refined and thus the grain size gradually decreased. Therefore, the mutual occurrence of grain boundary and dislocations pinning by the oxide particles (contribution to strengthening), and grains fragmentation providing restored grain boundaries available for grain boundary sliding (contribution to softening) finally resulted in the establishment of a steady state for the composites [73].

Regarding the differences between the consolidated structures, i.e., the *HRS* and *CRS* samples, the final swaging performed at the elevated temperature can be supposed to provide a structure featuring a higher plasticity compared to a structure finally swaged at room temperature, and thus also a lower flow stress [76]. However, the comparison of the experimentally acquired flow stress curves shown in Figure 6b,c reveals that the flow stress levels for both the *HRS* and *CRS* composites were comparable. In other words, no significant difference in the values of the flow stress between the *HRS* and *CRS* samples was observed, as both the *HRS* and *CRS* composites exhibited comparable deformation behaviors. Nevertheless, a slight difference could be seen at the peak of the curve—the *HRS* composite exhibited a plateau at the peak stress level and only after the applied true strain increased by ~0.1–0.2 (depending on the applied strain rate), the flow strain started to decrease. This decrease was then followed by the establishment of a steady state. On the other hand, the *CRS* composite exhibited an almost immediate flow stress decrease after achieving the stress peak, but the decrease before the establishment of the steady state was less steep than for the *HRS* composite. This phenomenon can again be explained with the help of the comparison of the structure states. The achieved average grain size for the *CRS* composite was lower than for the *HRS* one, and the oxide particles were also more fragmented (see Figure 3c,d and Figure 5c,d). Also, the grains within the final structure of the *HRS* composite exhibited a highly developed substructure, whereas the *CRS* composite exhibited restored UF grains after the final swaging. The LAGB fraction was slightly lower for the *CRS* sample, too. In accordance to this, the structure of the *HRS* composite needed a longer dwell time at the peak stress for the substructure to develop into newly formed refined grains, and subsequently exhibit grain boundary sliding [77]. On the other hand, the *CRS* composite structure featured restored UF grains without a significant presence of substructure, and thus the effect of grain boundary sliding was more immediate [78]. However, this was true only for a portion of the structure, as the structure still contained larger grains as well, which were subject to continuous fragmentation during the loading. This phenomenon resulted in a progressive increase in the extent of the occurrence of grain boundary sliding and consequent gradual establishment of a steady state [75]. In other words, by the effect of the mentioned phenomena, the decrease in flow stress with increasing true strain was more gradual for the *CRS* composite than for the *HRS* one.

**Figure 7:**
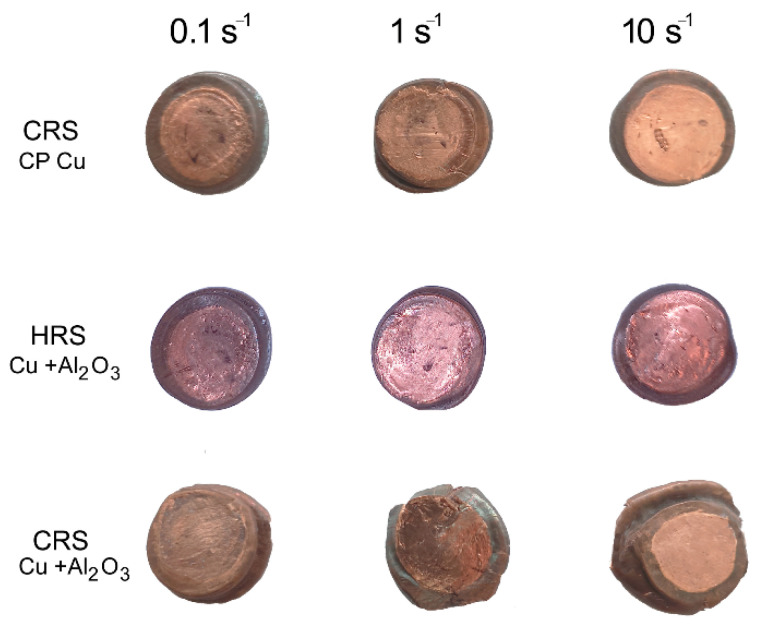
Test samples of examined materials after uniaxial compression tests.

The deformation behavior of the *CRS* composite was influenced by the presence of the Al_2_O_3_ particles more intensively than that of the *HRS* one. The reason for this more significant influence lies predominantly in the different plastic flow of the Cu matrix in the vicinity of the strengthening particles. In other words, the aggravated plasticity of the *CRS* composite (due to the lower processing temperature) must be taken into account when considering its plastic flow behavior. This factor is the primary cause of the sudden decrease in stress, as well as its following increase with increasing imposed strain. As a consequence of the natural brittleness of the Al_2_O_3_ particles, especially when processed at low/ambient temperatures, their more intense refinement, as well as more frequent interactions between the strengthening particles and Cu matrix, can be expected to occur within the *CRS* composite. On the other hand, the facilitated plastic flow of the *HRS* composite (compared to the *CRS* one) can be put in relation with the necessity of the Cu matrix to apply lower local forces on the Al_2_O_3_ particles. A greater loading of the particles brings about a higher probability of their fragmentation and subsequent direct influence of the fragments, i.e., newly originated barriers for the movements of dislocations on the plastic flow. Final instabilities of plastic flow can be seen for both the consolidation regimes, but more distinctly for the *CRS*. These conclusions were also supported by the plastic flow behavior; Figure 7, a photo of the tested samples in which it is depicted, documents that the plastic flow within the *HRS* composite was of a greater homogeneity than that of the *CRS* composite. The samples of the *CRS* composite exhibited non-circular shapes after the performed plastometric tests at all the examined strain rates. However, the tendency of the periphery of the samples to develop cracks evidently increased with increasing strain rate.

### 3.4. Electroconductivity

**Figure 8:**
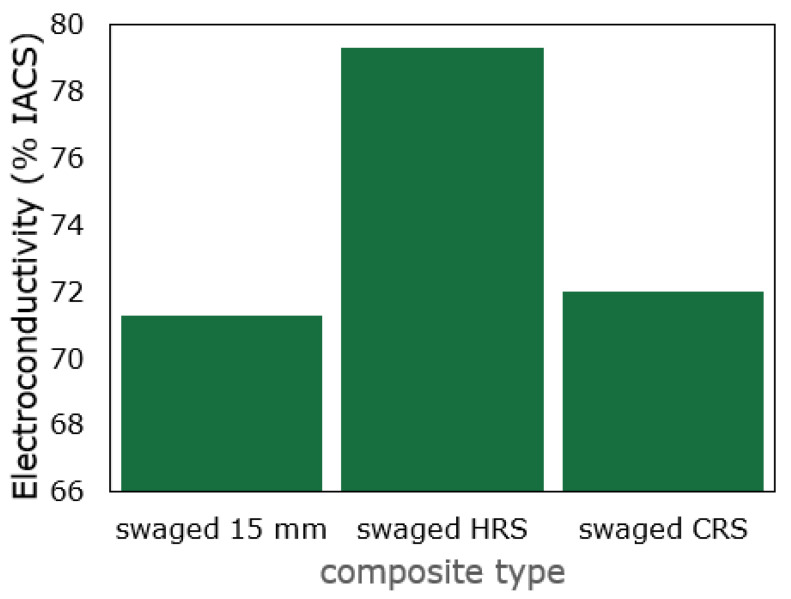
Experimentally measured electroconductivity for directly consolidated composites.

The electroconductivity of the directly consolidated composites was measured after swaging to the final diameter of 10 mm, as well as before differentiation of the swaging routes, i.e., after swaging to the diameter of 15 mm. The results of the measurements are summarized in Figure 8. Evidently, the electroconductivity decreased when compared to the electroconductive CP Cu in an annealed state, the standard IACS value for which is 100%. Among the examined consolidated composites, the measured electroconductivity was the lowest for the consolidated composite at the diameter of 15 mm. This fact can primarily be explained by the incomplete consolidation of the structure given by the relatively low applied imposed strain. The structure seemed to be well consolidated in the macroscale, but in the microscale, it contained local (micro)voids. Also, the structure featured an inhomogeneous grain size distribution and relatively large unfragmented oxide particles (see also Figure 2a), all of which contributed to the relatively high electric resistivity.

After increasing the processing temperature and continuing with the final swaging at 600 °C (i.e., the *HRS* composite), the electroconductivity increased to almost 80% IACS. This phenomenon most probably occurred since increasing the processing temperature supported the closing of internal voids and decrease in the residual porosity, as well as structure restoration and homogenization in regard to the grain size and its distribution (compare Figure 2a,b to Figure 3a,c). Decreasing the processing temperature during the final swaging to room temperature resulted only in marginal increase in the electroconductivity, and thus the final IACS electroconductivity value was lower for the *CRS* composite than for the *HRS* one. This can again be attributed to the differences in the structure states. Although both the 10 mm consolidated composites exhibited no presence of residual porosity, the *CRS* sample featured highly refined (UF) grains with fragmented oxides (see Figure 2b and Figure 5d), all of which acted as barriers for the free movement of electrons. In other words, the *HRS* sample exhibited a greater grain size, i.e., lower overall volume fraction of grain boundaries, as well as coarser oxide particles, by the mutual effect of which the electrons had more free space to move compared to the structure of the *CRS* composite. This conclusion is also supported by findings of Rajkovic et al. [57], who documented that the coarser the strengthening Al_2_O_3_ oxide particles are, the lower their deteriorating effect is on electroconductivity.

## 4. Conclusions

The herein presented study consisted of direct consolidation of a mixture of Cu + Al_2_O_3_ (5 wt. %) powders via rotary swaging, followed by investigations of the structure, deformation behavior, and electroconductivity. The processing route involved swaging at a temperature of 400 °C, and finally differentiated to swaging at 600 °C (*HRS* sample), and swaging at room temperature (*CRS* sample). The main acquired conclusions can be summarized as follows:

- the structures of both the *CRS* and *HRS* composites exhibited well-consolidated grains with the average areas of 1.2 µm^2^ and 1.8 µm^2^, respectively, and no significant preferential texture (especially the *CRS* sample);
- the *HRS* composite featured fine grains with a substructure, whereas the *CRS* one featured well-developed restored ultra-fine grains;
- the oxide particles were homogeneously distributed within both the composites, but within the *CRS* one they were finer and highly fragmented;
- the electroconductivity reached almost 80% IACS for the *HRS* sample;
- the plastic behavior was more favorable for the *CRS* sample (the stress-strain curves exhibited the establishment of a steady state with increasing strain).

The presented work proved that composites based on mixtures of Cu + Al_2_O_3_ powders can be successfully prepared by a direct consolidation via intensive plastic deformation, even at moderate and ambient temperatures. Our following research is going to be focused on the optimization of conditions of thermomechanical processing to increase the electroconductivity, while maintaining a favorable deformation behavior and mechanical properties.

## Figures and Tables

**Table 1:** Examined materials and applied compression test conditions.

Material	Sample	Applied Compression Test Conditions		
		Temperature (°C)	Strain Rate Range (s^−1^)	True Strain (-)
CP Cu	CRS	20	0.1–1–10	1.0
Cu + Al_2_O_3_	CRS	20	0.1–1–10	1.0
Cu + Al_2_O_3_	HRS	20	0.1–1–10	1.0

## Data Availability

The original data supporting the research are not publicly available but some of the data that are not confidential are available on request from the corresponding author.

## References

[1] Vityaz P.A., Ilyushchanka A.P., Savich V.V. (2019). Powder Metallurgy in Belarus and Global Developmental Trends. Russ. J. Non-Ferrous Met..

[2] Rojas-Díaz L.M., Verano-Jiménez L.E., Muñoz-García E., Esguerra-Arce J., Esguerra-Arce A. (2020). Production and Characterization of Aluminum Powder Derived from Mechanical Saw Chips and Its Processing through Powder Metallurgy. Powder Technol..

[3] Bharathi P., Kumar T.S. (2023). Mechanical Characteristics and Wear Behaviour of Al/SiC and Al/SiC/B4C Hybrid Metal Matrix Composites Fabricated through Powder Metallurgy Route. Silicon.

[4] Dewangan S.K., Nagarjuna C., Lee H., Sharma A., Ahn B. (2023). Surface Morphology Transformation and Densification Behaviour of Conventionally Sintered AlFeCoNiSi High Entropy Alloys. Powder Metall..

[5] Johnson J.L. (2023). Enhanced Sintering of Tungsten. Int. J. Refract. Met. Hard Mater..

[6] Yoon J.-W., Back J.-H. (2018). Effect of Sintering Conditions on the Mechanical Strength of Cu-Sintered Joints for High-Power Applications. Materials.

[7] Martínez C., Briones F., Rojas P., Aguilar C., Guzman D., Ordoñez S. (2017). Microstructural and Mechanical Characterization of Copper, Nickel, and Cu-Based Alloys Obtained by Mechanical Alloying and Hot Pressing. Mater. Lett..

[8] Rabiee M., Mirzadeh H., Ataie A. (2020). Mechanical Alloying and Consolidation of Copper-iron-silicon Carbide Nanocomposites. Mater. Werkst..

[9] Jamal N.A., Farazila Y., Ramesh S., Anuar H. (2014). Role of Mechanical Alloying Parameters on Powder Distribution of Al/Cu Alloy and Al/Cu Composite. Mater. Res. Innov..

[10] Seltzman A.H., Wukitch S.J. (2023). Precipitate Size in GRCop-42 and GRCop-84 Cu-Cr-Nb Alloy Gas Atomized Powder and L-PBF Additive Manufactured Material. Fusion Sci. Technol..

[11] Kunčická L., Kocich R., Németh G., Dvořák K., Pagáč M. (2022). Effect of Post Process Shear Straining on Structure and Mechanical Properties of 316 L Stainless Steel Manufactured via Powder Bed Fusion. Addit. Manuf..

[12] Rajaguru K., Karthikeyan T., Vijayan V. (2020). Additive Manufacturing—State of Art. Mater. Today Proc..

[13] Kunčická L., Macháčková A., Petrmichl R., Klečková Z., Marek M. (2020). Optimizing Induction Heating of WNiCo Billets Processed via Intensive Plastic Deformation. Appl. Sci..

[14] Macháčková A., Krátká L., Petrmichl R., Kunčická L., Kocich R. (2019). Affecting Structure Characteristics of Rotary Swaged Tungsten Heavy Alloy Via Variable Deformation Temperature. Materials.

[15] Kunčická L., Macháčková A., Lavery N.P., Kocich R., Cullen J.C.T., Hlaváč L.M. (2020). Effect of Thermomechanical Processing via Rotary Swaging on Properties and Residual Stress within Tungsten Heavy Alloy. Int. J. Refract. Met. Hard Mater..

[16] Sharma A., Zadorozhnyy M., Stepashkin A., Kvaratskheliya A., Korol A., Moskovskikh D., Kaloshkin S., Zadorozhnyy V. (2021). Investigation of Thermophysical Properties of Zr-Based Metallic Glass-Polymer Composite. Metals.

[17] Stef J., Poulon-Quintin A., Redjaimia A., Ghanbaja J., Ferry O., De Sousa M., Gouné M. (2018). Mechanism of Porosity Formation and Influence on Mechanical Properties in Selective Laser Melting of Ti-6Al-4V Parts. Mater. Des..

[18] Opěla P., Benč M., Kolomy S., Jakůbek Z., Beranová D. (2023). High Cycle Fatigue Behaviour of 316L Stainless Steel Produced via Selective Laser Melting Method and Post Processed by Hot Rotary Swaging. Materials.

[19] Jhunjhunwala P., Gupta A. (2023). Effect of Porosity on the Quality of 3D Printed Structures. Int. J. Adv. Manuf. Technol..

[20] Tusher M.M.H., Ince A. (2023). High Cycle Fatigue and Very High Cycle Fatigue Performance of Selective Laser Melting Ti-6Al-4V Titanium Alloy—A Review. Mater. Perform. Charact..

[21] Tamegai T., Pyon S., Ito T., Kajitani H., Koizumi N., Awaji S., Kito H., Ishida S., Yoshida Y. (2023). Fabrication of Small Magnets Using Mono- and Seven-Core (Ba, A)Fe 2 As 2 (A : K, Na) HIP Round Wires. IEEE Trans. Appl. Supercond..

[22] Carvajal A.H.R., Ríos J.M., Zuleta A.A., Bolívar F.J., Castaño J.G., Correa E., Echeverria F., Lambrecht M., Lasanta M.I., Trujillo F.J.P. (2023). Development of Low Content Ti-X%wt. Mg Alloys by Mechanical Milling plus Hot Isostatic Pressing. Int. J. Adv. Manuf. Technol..

[23] Staab F., Bruder E., Schäfer L., Skokov K., Koch D., Zingsem B., Adabifiroozjaei E., Molina-Luna L., Gutfleisch O., Durst K. (2023). Hard Magnetic SmCo5-Cu Nanocomposites Produced by Severe Plastic Deformation. Acta Mater..

[24] Evdokimov I.A., Khayrullin R.R., Bagramov R.K., Perfilov S.A., Pozdnyakov A.A., Aksenenkov V.V., Kulnitskiy B.A. (2021). Nanostructured Strain-Hardened Aluminum–Magnesium Alloys Modified by C60 Fullerene Obtained by Powder Metallurgy: 2. The Effect of Magnesium Concentration on Physical and Mechanical Properties. Russ. J. Non-Ferrous Met..

[25] Huang M., Jiang J., Wang Y., Liu Y., Zhang Y., Dong J., Xiao G. (2022). Deformation Behavior, Microstructure Evolution, Phase Transformation and Plastic Instability Origin of Powder Metallurgy Al0.8Co0.5Cr1.5CuFeNi Alloy during High Temperature Deformation. Mater. Sci. Eng. A.

[26] Orlov D., Lapovok R., Toth L.S., Timokhina I.B., Hodgson P.D., Haldar A., Bhattacharjee D. (2014). Asymmetric Rolling of Interstitial-Free Steel Using Differential Roll Diameters. Part II: Microstructure and Annealing Effects. Metall. Mater. Trans. A.

[27] Hedicke-Claus Y., Kriwall M., Stonis M., Behrens B.-A. (2023). Automated Design of Multi-Stage Forging Sequences for Die Forging. Prod. Eng..

[28] Cvijović Z., Rakin M., Vratnica M., Cvijović I. (2008). Microstructural Dependence of Fracture Toughness in High-Strength 7000 Forging Alloys. Eng. Fract. Mech..

[29] Haase M., Tekkaya A.E. (2015). Cold Extrusion of Hot Extruded Aluminum Chips. J. Mater. Process. Technol..

[30] Peretyat’ko V.N., Smetanin S.V. (2016). Energy-Efficient Four-Roll Rail Rolling Technology. Metallurgist.

[31] Kunčická L., Klečková Z. (2020). Structure Characteristics Affected by Material Plastic Flow in Twist Channel Angular Pressed Al/Cu Clad Composites. Materials.

[32] Kocich R., Kunčická L. (2021). Development of Structure and Properties in Bimetallic Al/Cu Sandwich Composite during Cumulative Severe Plastic Deformation. J. Sandw. Struct. Mater..

[33] Vargas M., Lathabai S., Uggowitzer P.J., Qi Y., Orlov D., Estrin Y. (2017). Microstructure, Crystallographic Texture and Mechanical Behaviour of Friction Stir Processed Mg-Zn-Ca-Zr Alloy ZKX50. Mater. Sci. Eng. A.

[34] Kunčická L., Kocich R., Drápala J., Andreyachshenko V.A. FEM Simulations and Comparison of the Ecap and ECAP-PBP Influence on Ti6Al4V Alloy’s Deformation Behaviour. Proceedings of the METAL 2013 22nd International Conference on Metallurgy and Materials.

[35] Jamili A.M., Zarei-Hanzaki A., Abedi H.R., Mosayebi M., Kocich R., Kunčická L. (2019). Development of Fresh and Fully Recrystallized Microstructures through Friction Stir Processing of a Rare Earth Bearing Magnesium Alloy. Mater. Sci. Eng. A.

[36] Kunčická L., Kocich R., Král P., Pohludka M., Marek M. (2016). Effect of Strain Path on Severely Deformed Aluminium. Mater. Lett..

[37] Liang W., Bian L., Xie G., Zhang W., Wang H., Wang S. (2010). Transformation Matrix Analysis on the Shear Characteristics in Multi-Pass ECAP Processing and Predictive Design of New ECAP Routes. Mater. Sci. Eng. A.

[38] Şimşir C., Karpuz P., Gür C.H. (2010). Quantitative Analysis of the Influence of Strain Hardening on Equal Channel Angular Pressing Process. Comput. Mater. Sci..

[39] Martynenko N.S., Bochvar N.R., Rybalchenko O.V., Bodyakova A.I., Morozov M.M., Leonova N.P., Yusupov V.S., Dobatkin S.V. (2022). Effect of Rotary Swaging and Subsequent Aging on the Structure and Mechanical Properties of a Cu–0.5% Cr–0.08% Zr Alloy. Russ. Metall..

[40] Estrin Y., Martynenko N., Lukyanova E., Serebryany V., Gorshenkov M., Morozov M., Yusupov V., Dobatkin S. (2020). Effect of Rotary Swaging on Microstructure, Texture, and Mechanical Properties of a Mg-Al-Zn Alloy. Adv. Eng. Mater..

[41] Panov D., Kudryavtsev E., Naumov S., Klimenko D., Chernichenko R., Mirontsov V., Stepanov N., Zherebtsov S., Salishchev G., Pertcev A. (2023). Gradient Microstructure and Texture Formation in a Metastable Austenitic Stainless Steel during Cold Rotary Swaging. Materials.

[42] Droste M., Ullrich C., Motylenko M., Fleischer M., Weidner A., Freudenberger J., Rafaja D., Biermann H. (2018). Fatigue Behavior of an Ultrafine-Grained Metastable CrMnNi Steel Tested under Total Strain Control. Int. J. Fatigue.

[43] Wang Z., Chen J., Besnard C., Kunčická L., Kocich R., Korsunsky A.M. (2021). In Situ Neutron Diffraction Investigation of Texture-Dependent Shape Memory Effect in a near Equiatomic NiTi Alloy. Acta Mater..

[44] Kunčická L., Kocich R. (2022). Effect of Activated Slip Systems on Dynamic Recrystallization during Rotary Swaging of Electro-Conductive Al-Cu Composites. Mater. Lett..

[45] Rogachev S.O., Sundeev R.V., Andreev V.A., Andreev N.V., Tabachkova N.Y., Korotkova N.O. (2022). The Microstructure and Conductivity of Copper–Aluminum Composites Prepared by Rotary Swaging. Phys. Met. Metallogr..

[46] Giribaskar S., Gouthama, Prasad R. (2011). Ultra-Fine Grained Al-SiC Metal Matrix Composite by Rotary Swaging Process. Mater. Sci. Forum.

[47] Tian W., Zhang F., Han S., Chen X., Gao P., Zheng K. (2023). Analysis of Microstructure and Properties in Cold Rotary Swaged Copper-Clad Magnesium Wires. Metals.

[48] Chen C., Wang W., Guo Z., Sun C., Volinsky A.A., Paley V. (2018). Annealing Effects on Microstructure and Mechanical Properties of Ultrafine-Grained Al Composites Reinforced with Nano-Al2O3by Rotary Swaging. J. Mater. Eng. Perform..

[49] Seixas M.R., Bortolini C., Pereira A., Nakazato R.Z., Popat K.C., Alves Claro A.P.R. (2018). Development of a New Quaternary Alloy Ti–25Ta–25Nb–3Sn for Biomedical Applications. Mater. Res. Express.

[50] Chi F., Wießner L., Gröb T., Bruder E., Sawatzki S., Löwe K., Gassmann J., Müller C., Durst K., Gutfleisch O. (2019). Towards Manufacturing of Nd-Fe-B Magnets by Continuous Rotary Swaging of Cast Alloy. J. Magn. Magn. Mater..

[51] Kataoka K., Matsuura M., Tezuka N., Sugimoto S. (2015). Influence of Swaging on the Magnetic Properties of Zn-Bonded Sm-Fe-N Magnets. Mater. Trans..

[52] Rogachev S.O., Andreev V.A., Gorshenkov M.V., Ten D.V., Kuznetsova A.S., Shcherbakov A.B. (2022). Rotary Forging to Improve the Strength Properties of the Zr–2.5% Nb Alloy. Phys. Met. Metallogr..

[53] Martynenko N., Rybalchenko O., Bodyakova A., Prosvirnin D., Rybalchenko G., Morozov M., Yusupov V., Dobatkin S. (2022). Effect of Rotary Swaging on the Structure, Mechanical Characteristics and Aging Behavior of Cu-0.5%Cr-0.08%Zr Alloy. Materials.

[54] Martynenko N., Anisimova N., Kiselevskiy M., Tabachkova N., Temralieva D., Prosvirnin D., Terentiev V., Koltygin A., Belov V., Morosov M. (2020). Structure, Mechanical Characteristics, Biodegradation, and in Vitro Cytotoxicity of Magnesium Alloy ZX11 Processed by Rotary Swaging. J. Magnes. Alloy..

[55] Svoboda J., Kunčická L., Luptáková N., Weiser A., Dymáček P. (2020). Fundamental Improvement of Creep Resistance of New-Generation Nano-Oxide Strengthened Alloys via Hot Rotary Swaging Consolidation. Materials.

[56] Gnanasambandam P., Kumar A., Nandy T.K. (2018). Effect of Yttrium Oxide Dispersion on the Microstructure and Properties of Tungsten Heavy Alloys. Def. Sci. J..

[57] Hupalo M.F., Padilha A.F., Sandim H.R.Z., Kliauga A.M. (2004). Cold Swaging, Recovery and Recrystallization of Oligocrystalline INCOLOY MA 956-Part I: Deformed State. ISIJ Int..

[58] Mateus R., Carvalho P.A., Nunes D., Alves L.C., Franco N., Correia J.B., Alves E. (2011). Microstructural Characterization of the ODS Eurofer 97 EU-Batch. Fusion Eng. Des..

[59] Russell A., Lee K.L. (2005). Structure-Property Relations in Nonferrous Metals.

[60] Miyoshi H., Kimizuka H., Ishii A., Ogata S. (2021). Competing Nucleation of Single- and Double-Layer Guinier–Preston Zones in Al–Cu Alloys. Sci. Rep..

[61] Kashyap K.T., Koppad P.G. (2011). Small-Angle Scattering from GP Zones in Al-Cu Alloy. Bull. Mater. Sci..

[62] Fukamachi K. (2023). Detailed Relationship between the Microstructure and Properties of Age-Hardened Cu–4 At% Ti Alloy. Mater. Today Commun..

[63] Lomakin I., Nigmatullina A., Sauvage X. (2021). Mechanism of Large Strain Accommodation Assisted by Shear Localization in a Precipitation-Hardened Cu–Be Alloy. Mater. Sci. Eng. A.

[64] Jiang Y., Zhang X., Cai P., Li P., Cao F., Gao F., Liang S. (2023). Precipitation Behavior and Microstructural Evolution during Thermo-Mechanical Processing of Precipitation Hardened Cu-Hf Based Alloys. Acta Mater..

[65] Carneiro Í., Monteiro B., Ribeiro B., Fernandes J.V., Simões S. (2023). Production and Characterization of Cu/CNT Nanocomposites. Appl. Sci..

[66] Moustafa S., Abdel-Hamid Z., Abd-Elhay A. (2002). Copper Matrix SiC and Al2O3 Particulate Composites by Powder Metallurgy Technique. Mater. Lett..

[67] Marzun G., Bönnemann H., Lehmann C., Spliethoff B., Weidenthaler C., Barcikowski S. (2017). Role of Dissolved and Molecular Oxygen on Cu and PtCu Alloy Particle Structure during Laser Ablation Synthesis in Liquids. ChemPhysChem.

[68] Jeyaprakash N., Kumar M.S., Yang C.-H. (2023). Enhanced Nano-Level Mechanical Responses on Additively Manufactured Cu-Cr-Zr Copper Alloy Containing Cu2O Nano Precipitates. J. Alloys Compd..

[69] Rajkovic V., Bozic D., Jovanovic M.T. (2008). Properties of Copper Matrix Reinforced with Various Size and Amount of Al2O3 Particles. J. Mater. Process. Technol..

[70] Feng J., Song K., Liang S., Guo X., Li S. (2022). Mechanical Properties and Electrical Conductivity of Oriented-SiC-Whisker-Reinforced Al_2_O_3_/Cu Composites. J. Mater. Res. Technol..

[71] Kocich R., Kunčická L. (2023). Optimizing Structure and Properties of Al/Cu Laminated Conductors via Severe Shear Strain. J. Alloys Compd..

[72] Kunčická L., Kocich R. (2022). Optimizing Electric Conductivity of Innovative Al-Cu Laminated Composites via Thermomechanical Treatment. Mater. Des..

[73] Humphreys F.J., Hetherly M., Rollett A., Rohrer G.S. (2004). Recrystallization and Related Annealing Phenomena.

[74] Freudenberger M., Vernes A., Fotiu P.A. (2023). An Analytical Model of Brinell Hardness for Power-Law Hardening Materials. Results Eng..

[75] Verlinden B., Driver J., Samajdar I., Doherty R.D. (2007). Thermo-Mechanical Processing of Metallic Materials.

[76] Canelo-Yubero D., Kocich R., Hervoches C., Strunz P., Kunčická L., Krátká L. (2021). Neutron Diffraction Study of Residual Stresses in a W–Ni–Co Heavy Alloy Processed by Rotary Swaging at Room and High Temperatures. Met. Mater. Int..

[77] Langdon T.G. (2006). Grain Boundary Sliding Revisited: Developments in Sliding over Four Decades. J. Mater. Sci..

[78] Sharififar M., Akbari Mousavi S.A.A. (2014). Tensile Deformation and Fracture Behavior of CuZn5 Brass Alloy at High Temperature. Mater. Sci. Eng. A.

